# An exotic abscess within the United Kingdom from The Gambia: a case report

**DOI:** 10.1186/s13256-017-1472-3

**Published:** 2017-11-03

**Authors:** Estelle Hong How, Darren Yap, Nik Mbakada

**Affiliations:** Department of Emergency medicine, Royal Blackburn Hospital, Haslingden Road, Blackburn, Lancashire BB2 3HH UK

**Keywords:** Tumbu fly, Gambia, Furuncular myiasis, *Cordylobia anthropophaga*

## Abstract

**Background:**

Furuncular myiasis is a parasitic infection of a live mammal by fly larvae commonly seen in Africa. However, with an increase in international tourism, there is a significant rise in exotic infection in non-endemic areas which can pose a diagnostic challenge to doctors and potentially lead to delay in treatment. From the current literature, only 12 cases were reported in the UK.

**Case presentation:**

We report an unusual case of multiple abscesses in a 32-year-old white British woman presenting to our Emergency department in the UK after returning from a holiday in The Gambia, West Africa. She did not complain of systemic symptoms and was otherwise fit and healthy with no significant past medical history. During examination, two maggots were expressed from the abscesses by applying lateral pressure to each lesion. The larvae were found to be *Cordylobia anthropophaga*. She was discharged with antibiotics to prevent secondary infection with no further follow-up.

**Conclusion:**

With globalization, the need for increasing awareness of tropical diseases has become important to win the battle against future epidemics.

## Background

Furuncular myiasis is a parasitic infestation of the body of a live mammal by fly larvae of dipterous insects that feed on their host’s tissue to grow and mature. This condition is only prevalent in tropical countries where these flies are common; these flies normally do not thrive in the United Kingdom (UK) [1, 2]. However, with globalization and the prodigious advancement in transportation, there is an increase in the number of reported cases of furuncular myiasis in the UK [1, 3]. This exotic infestation can in fact pose a significant diagnostic challenge for clinicians. In this era, where exotic infections could potentially lead to catastrophic life-threatening epidemics, it is important to take a good travel history. It is extremely important to have an awareness of disease prevalence in the areas of travel and the ability to access up-to-date information [1, 2]. To the best of our knowledge, only 12 cases of furuncular myiasis by the tumbu fly have been reported in the UK and we present the 13th case of this unfamiliar condition [4–7] (Table 1).

**Table 1:** Reported cases of furuncular myiasis within the current literature

Characteristics of furuncular myiasis in returning travelers in the United Kingdom									
Article	Study year	Number of cases	Country of origin	Gender	Age (years)	Number of lesions	Number of extracted maggots	Location of lesions	Symptoms
McGarry *et al*. [4]	1994–2000	6	The Gambia	*	*	*	*	*	*
		2	Nigeria	*	*	*	*	*	*
		1	Equatorial Guinea	*	*	*	*	*	*
Lee and Robinson [5]	2006	1	Angola	Male white	39	1 + (unreported number of lesions)	1	Face, thigh, buttocks	Pain, swelling, erythema
James and Stevenson [6]	1992	1	Nairobi	Male	26	12	12	Mid-scapular region	Pain, itch
Whitehorn *et al*. [7]	2009	1 (via household contact)	Uganda	Male	40	1	1	Right forearm	Itch, swelling, erythema

*unreported

*UK* United Kingdom

## Case presentation

A 32-year-old white woman presented to her general practitioner (GP) with a 1-week history of two thigh lumps and another on her left flank. She first noticed these lumps a week after returning from her holiday in The Gambia. She was otherwise well with no significant past medical history. Her GP had treated her as having multiple skin abscesses and started her on a course of antibiotic therapy (amoxicillin). Two days later, she expressed two live maggots from the thigh abscesses which prompted her to visit our Emergency department (ED).

**Fig. 1:**
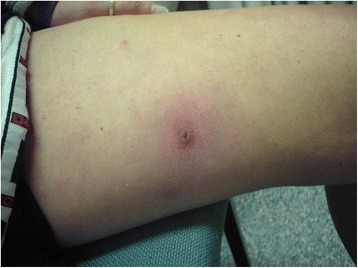
A 2 cm in diameter abscess on the right thigh with a central punctum surrounded by an erythematous ring

**Fig. 2:**
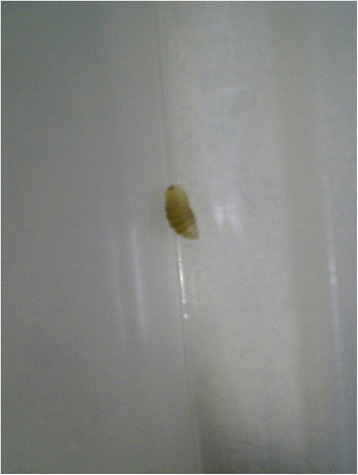
A live maggot expressed from left flank region

On initial examination she was systemically well and apyrexial. Two abscesses, each measuring approximately 2 × 2 cm were noted on her right thigh and one on her left flank. The lesions had a punctum surrounded by a ring of erythema with no obvious discharge as shown in Fig. 1. There was no evidence of tracking cellulitis or lymphadenopathy. A live maggot approximately 6 mm long was expressed when pressure was applied to her flank lesion as shown in Fig. 2.

She was discharged home on a course of flucloxacillin (1 g, four times a day for 7 days) and a follow-up appointment was arranged. We contacted The Liverpool School of Tropical Medicine for advice and the African tumbu fly was suggested to be the culprit. No formal entomology examination was carried out and microbiology culture samples yielded no growth after 48 hours. The Liverpool School of Tropical Medicine recommended covering the lesions with Vaseline (petroleum jelly) and occlusive dressing to suffocate any further larvae and replacing flucloxacillin with co-amoxiclav. Our patient was seen 2 days later and the diagnosis was explained. The wounds were healing remarkably and she was discharged with an antibiotic course of co-amoxiclav (500/125 mg tablet, three times a day for 7 days) with no further follow-up required.

## Discussion

### What is furuncular myiasis?

Cases of myiasis affecting humans have been described in the literature as far back as 1840 [8]. Myiasis is derived from the ancient Greek word for fly: “Myia.” Furuncular myiasis is a cutaneous parasitic infection caused by a particular species of Diptera flies called *Cordylobia anthropophaga,* also known as the African tumbu fly, giving rise to “boil” (furuncular)-like lesions [9, 10].

### Epidemiology

It is a common condition in the equatorial regions of Africa. It infests mainly animals such as dogs and wild rats which constitute an important reservoir of the infection to humans. In this part of the world, the disease spreads widely due to animal migration and rainy seasons [1, 2, 10, 11]. Myiasis in tropical Africa is predominantly caused by tumbu fly larvae [10, 12]. In Africa, there is a higher incidence in children compared to adults due to the fact that children have thinner skin which is more easily penetrated by the larvae [1, 2]. In a 7-year study conducted by experts at The Liverpool School of Tropical Medicine and Hygiene from 1994 to 2000, in order to determine the epidemiology of ectoparasitic diseases in returning travelers, where 73 specimens were studied; 18 cases (67%) of myiasis were more common in returning travelers in comparison to 6 cases (33%) infected within the UK [4]. Furuncular myiasis larval infestation by tumbu fly (*n* = 9, 50%) in travelers returning was the predominant cause of myiasis. The commonest countries visited by those infected were The Gambia (*n* = 6), Nigeria (*n* = 2), and Equatorial Guinea (*n* = 1). It was concluded by the authors that exotic infection especially myiasis, predominated in returning travelers from Africa and Latin America [4].

### The life cycle of the tumbu fly

The tumbu fly (*Cordylobia anthropophaga*), a large brown yellowish fly, is endemic in the subtropics of Africa. The larvae feed and develop in the host’s tissue, usually the skin or body orifices [13]. The female fly has a short life span of 2 to 3 weeks and deposits between 100 and 500 eggs in sand. Occasionally, eggs can be found on sweat-soaked clothes or on babies’ soiled napkins [1, 7]. This explains why the lesions are most often found on covered sites such as buttocks, trunk, or thighs [2]. The eggs take between 1 and 3 days to hatch into first-stage larvae measuring approximately 1 mm in length and are able to survive without food for approximately 9 to 15 days. During this time, they remain dormant under a layer of sand [1, 10].

The young larvae creep to the surface of the sand; with their upper bodies projecting above the surface they can reach and burrow under the host’s skin for refuge. This process is usually innocuous and hardly noticed [1, 2, 14]. Occasionally, the larvae can gain access to humans when infected clothes are worn [7]. The speed of penetration depends greatly on the thickness of the skin. Hence, children or young animals with thinner skin are more commonly affected. The larvae do not generally migrate past the dermis [14]. The affected skin initially turns into an erythematous papule of a few millimeters in diameter [1]. The immature larvae feed on the host’s tissue and develop into secondary then tertiary mature larvae in 8 to 10 days.

During this time, the papule has developed into an inflamed tender and edematous abscess-like furuncle approximately 2 cm in diameter surrounded by an erythematous ring. An ulcer or central punctum is formed at the top of the lesion whereby the posterior end of the larvae allows respiration. Sometimes, the spiracle of the larvae or a rhythmical movement can be seen through this central depression. On some occasions, the white body of the larvae can also be seen projecting from the lesion [1, 2, 13–16]. The larvae normally exit the host through the breathing pore after approximately 8 to 15 days to become hard pupae that become adult flies in a period of 3 to 4 weeks [2, 5, 10, 14].

### Clinical presentation

In humans, the most common sites affected are the neck, back, and trunk. Other common sites of infection are the scrotum in men and breast in women [1, 10, 13, 17]. To the best of our knowledge, less than ten larvae are usually extracted in an infected patient. However, up to 64 maggots were retrieved from a white soldier based in West Africa and 94 from a child in Ghana [14, 18].

The symptoms, which usually develop within 2 days of infestation, can vary from being asymptomatic to very severe [15]. The lesions are usually tender and pruritic but ultimately benign. In general, patients do not have systemic symptoms such as fever or malaise [5, 13, 15–17]. A few cases were reported where patients had fever [10, 19, 20, 21]. This was the case when there were multiple infestations and subsequently there was an increase in systemic inflammatory markers, eosinophils, and immunoglobulin E level [2, 10, 19, 18]. Sometimes, the pain and itch could not be borne, which led to extreme agitation, insomnia, and distress in the patient [6, 14, 18]. As a result, some patients were diagnosed as having delusional parasitosis due to the sensation of movement or crawling under the skin [2, 14].

Differential diagnoses include bacterial furunculosis, actinomycosis, chronic ulcer [13], staphylococcal infections, sebaceous cyst, cat-scratch disease, tick-bite granuloma, tungiasis (infestation by a burrowing flea), insect bite, pyoderma, mycosis, furuncular breast lesion from tuberculosis, fungating malignancies, and infestation with various parasitic worms (such as *Dirofilaria, Loa loa,* and *Onchocerca*) [13, 16, 22]. *Cordylobia* infestation can sometimes mimic severe skin infection such as herpes zoster or cellulitis especially when numerous lesions coalesce to form an erythematous and tender plaque-like rash [2, 14].

### Investigations and diagnosis

Extraction of the larva confirms the diagnosis of myiasis. Identification of the larvae can be undertaken through microscopic analysis of their external body form and the morphology of their characteristic posterior pair of respiratory spiracle slits used to obtain oxygen [1].

Ultrasound with high frequency linear transducer for better resolution to detect movement of larvae is useful to provide a definite diagnosis. The larva is perceived on ultrasound as a superficial encapsulated hyperechoic object with shadowing within the skin tissue. This is opposite to the finding of an abscess which will show on ultrasound an irregular collection of hypoechoic pus under the skin. The use of color Doppler sonography to view the movement of the fluid inside the larvae is useful to detect the parasite when ultrasound fails to do so [2, 20]. In Japan, in 2008, molecular analysis (that is, polymerase chain reaction (PCR)) was used to identify the species of the larvae, hence, allowing correct diagnosis with minimum experience of tropical infection [15].

Clinical laboratory results are usually normal except in severe multiple infestations, then mild eosinophilia is usually reported. No biopsy is required for diagnosis [2, 10, 16, 18]. However, knowledge of skin lesion and a good travel history with particular emphasis on specific location, timing, and risk behavior, and physical examination would lead to a correct diagnosis and simple treatment as outlined below. Any unhealed skin lesion in returning travelers from Africa and their household contacts should prompt the possibility of myiasis [7, 12].

### Treatment

Definitive treatment aims at extracting the larva in its third stage and treating any associated infection with antibiotics [23]. Suffocation of the larvae especially those in their second or third stage of development by application of Vaseline (petroleum jelly), petroleum jelly, liquid paraffin, or sticking adhesive tape seemed to be the most effective and less invasive treatment. This may force larvae to wriggle out as they will gradually move backwards towards the surface in search of oxygen. At the same time, the cavity becomes lubricated further loosening the grip of the larvae to the wall of the cavity facilitating mechanical extraction using an aseptic technique. The process may be aided by a gentle squeeze downwards and inwards [1, 2, 7, 10, 14, 24, 25]. Sometimes, hypoxia forces the larvae out enough for a doctor to use forceps to remove the rest of the body. Infiltration of the lesion with lidocaine and adrenaline or liquid nitrogen seemed to aid in the expulsion of the larvae as it acts as a paralyzing agent [2, 5, 20].

The danger of suffocating the larvae is that the process is slow and can take up to 24 hours. During this time, the larvae may fail to wriggle out and die of hypoxia [2]. Consequently, surgical incision and drainage may be necessary when asphyxiation technique fails or to extract the dead or decaying larvae [10, 14, 18]. It is crucial to avoid rupturing a larva during extraction because a remnant may cause an intense granulomatous inflammation and complicate the wound [7, 16]. Some lesions may also contain more than one larva [14]. In Germany, the innovative technique of using hydraulic pressure alone to extract the larvae was used successfully in two cases [21] but the effectiveness of this type of technique is yet to be tested on a larger scale.

Serious complications are very rare with the African tumbu fly and include tetanus, cellulites, osteomyelitis, and pyomyositis [13]. Sometimes with several larvae infestations close in proximity, shedding of dead skin and gangrene may occur [10]. In fact, secondary pyogenic infection is uncommon due to the bacteriostatic activity of the larvae in the skin. The release of phenylacetic acid and phenylacetaldehyde from the larvae’ gut suppresses the growth of bacteria [16, 26]. After the extraction of the larvae, the wound usually heals spontaneously leaving only some pigmented or depigmented lesions [13, 17]. Sometimes, disinfectant agents such as iodine and ethanol can be used to prevent secondary infections [10].

### Prevention

People traveling to endemic areas with furuncular myiasis should be educated at travel clinics of the effective measures to prevent such infection. Effective measures include the careful ironing of all clothing and bedding that has been dried outside, paying close attention to the seams. Travelers should maintain good personal hygiene and avoid being in contact with animals such as dogs and rodents as they are the natural hosts of the larvae. If possible, clothing should be dried in full sunlight, off the ground, and in a ventilated area under a mosquito net. Travelers should also avoid sleeping or sunbathing on the ground. Lastly, insecticides can be used to eliminate flies in living space or work place [5, 8, 10, 12, 13, 16–18].

### Management/follow-up

Usually no follow-up is required because complications are rare. Patients are prescribed a course of antibiotics to prevent secondary infections. However, in some cases, infestations can cause great psychological distress that requires counseling [5–7, 14].

## Conclusions

Despite being very common in Africa, furuncular myiasis by tumbu fly is a rare condition encountered by doctors in other countries. Subsequently, most practitioners are unfamiliar with the condition. However, with globalization and faster means of travel, an increased incidence has been reported in non-endemic countries. In most cases reported, the condition was quite often misdiagnosed for other infections, for example herpes zoster, impetigo, or folliculitis, consequently, causing undue distress in the patient [12, 14]. In addition, antibiotics and other unnecessary medications were often prescribed to patients with no results. This highlights the importance of better awareness of tropical infections to avoid any misdiagnosis, delay in diagnosis, or inappropriate treatment [7, 13, 18, 20].
