# Correction to: An exotic abscess within the United Kingdom from The Gambia: a case report

**DOI:** 10.1186/s13256-018-1565-7

**Published:** 2018-01-31

**Authors:** Estelle How-Hong, Darren Yap, Nik Mbakada

**Affiliations:** Department of Emergency medicine, Royal Blackburn Hospital, Haslingden Road, Blackburn, Lancashire BB2 3HH UK

## Correction

In the publication of this article [1], there is an error in an authors name.

The error: ‘Estelle Hong How‘ Should instead read: ‘Estelle How-Hong‘

This has now been included in this erratum.
